# Within-person face recognition strongly correlates with objective face processing assessments: a study beyond the WEIRD populations

**DOI:** 10.1098/rsos.250998

**Published:** 2025-11-12

**Authors:** Majeed Ali, Sarina Hui-Lin Chien

**Affiliations:** ^1^ Graduate Institute of Biomedical Sciences, China Medical University, Taichung City, Taiwan; ^2^ Center for Neuroscience and Brain Disease, China Medical University, Taichung City, Taiwan

**Keywords:** face perception, within-person variability, Face Identity Sorting task, Cambridge Face Memory Test, PI-20 questionnaire

## Abstract

Familiarity plays a pivotal role in within-person face recognition. However, previous research in this field mostly used only one task: Identity Card Sorting. This study aims to explore the links between within-person recognition and other standardized face processing tests, and to extend the sample beyond the WEIRD (western, educated, industrialized, rich and democratic) population. We recruited Taiwanese, Pakistanis and another group of international adults who were neither Taiwanese nor Pakistanis. Each participant completed Face Identity Sorting, Face Discrimination, the Cambridge Face Memory Test (CFMT) and the Prosopagnosia Index (PI-20). Results showed a significant familiarity effect in Face Identity Sorting, with Taiwanese and Pakistani groups, but not the International group, sorting fewer piles for familiar faces (i.e. Pakistani or Taiwanese celebrities). Correlational analyses combining all participants further revealed that Card Sorting performance positively correlated with CFMT and Face Discrimination scores, and showed a marginally negative correlation with PI-20. This suggests that individuals with better face memory and detail-oriented discriminability tend to make fewer errors in the unfamiliar within-person recognition task. This study is the first to examine within-person face recognition in Pakistani and Taiwanese adults, demonstrating a robust effect of familiarity and strong associations with objective assessments of face processing abilities.

## 1. Introduction

Face recognition is a vital cognitive skill that enables us to accurately discern identity, sex, race and age, playing a crucial role in our daily lives [1–4]. Previous research has primarily focused on between-person face recognition, often relying on single images to represent identity. However, this approach overlooks the significant challenge of within-person variability—differences in an individual’s appearance caused by biological, physiological and environmental factors [4,5]. In 2011, Jenkins *et al.* [5] developed a novel card sorting paradigm using ambient photos to investigate within-person recognition. They found that UK participants who were unfamiliar with the Dutch celebrities sorted a single person into several identities, whereas Dutch participants who were familiar with the celebrities sorted them into two identities perfectly. In other words, recognizing unfamiliar faces across different images can be challenging; people often mistake photos of the same person for those of different individuals. By contrast, familiarity greatly improves recognition accuracy, suggesting that a stable representation in memory lessens the negative effects of variations in appearance [5,6].

The importance of familiarity in face processing has been well established. Bruce & Young’s [1] face recognition model emphasized distinct routes and detailed processes for recognizing familiar and unfamiliar faces. Supporting this model, Megreya & Burton [7] found that familiar and unfamiliar face processing only correlates strongly when faces are inverted, indicating fundamentally different identity processing mechanisms for familiar versus unfamiliar faces. In addition, familiarity with faces enhances recognition accuracy under degraded viewing conditions. For example, people struggled to recognize unfamiliar faces in low-quality images, but could easily identify familiar faces under similar conditions [8–10]. Similarly, Burton *et al*. [11] demonstrated that observers can recognize faces more effectively, even in low-quality surveillance videos, when the faces belong to familiar individuals rather than those with whom they rarely interact. To address the mechanism underlying the familiarity effect, Kramer *et al*. [12] proposed that repeated exposure enhances memory representations of familiar faces, facilitating identification by promoting reliance on stable internal features, such as the eyes and mouth. They argued that familiarity reflects increasingly robust statistical representations of idiosyncratic within-person variability, supported by both bottom-up and top-down processes that aid in integrating fragmented facial features information.

The major paradigm of the card sorting task that examines within-person face recognition uses full-view face photographs of familiar individuals or well-known culture-specific celebrities [5,13–15]. Recent studies have expanded the card sorting paradigm and consistently found the robust effects of familiarity. For example, Balas & Pearson [16] introduced two new aspects to this card sorting paradigm. First, they used images of four Dutch female celebrities, including face-only, body-only, and both face and body photos. Second, in addition to the number of piles and the number of misidentification errors used in previous studies, they further introduced sensitivity (d') measure as a combined index of performance that captured two types of errors: ‘different person/same group’ errors (grouping images of different people in the same pile) and ‘same person/different group’ errors (separating the images of the same person into different piles). Their result showed that, while sorting images of unfamiliar individuals, participants sorted a single person into several identities, particularly when only facial information was available. Interestingly, recognition improved when both face and body cues were present. Kramer *et al*. [12] further examined the importance of internal and external features in familiar and unfamiliar face recognition, using a full face along with only internal or external features. They revealed that people accurately recognize faces when they are familiar with full view or when only the internal features (i.e. the eyes and mouth) are visible, while external features (i.e. hair and face shape) are less helpful. This suggests that the most essential details for identifying faces come from the inner parts of the face, regardless of whether the person is familiar or unfamiliar. Recently, Strathie *et al*. [17] extended this line of research by investigating whether the benefits of familiarity extend to recognizing siblings. They demonstrated that familiarity with one sibling enhances kin detection, as individuals more accurately recognized the siblings of familiar celebrities than those of unfamiliar ones. Unfamiliar face-matching performance was also higher when targets were the siblings of familiar individuals, even in the absence of the familiar sibling. This suggests that prior experience facilitates the recognition of related faces, indicating that familiarity improves identity matching and extends to kinship detection among unfamiliar faces.

Familiarity may operate on two levels: the personal level, where individuals recognize certain faces based on their own experiences with those people, and the racial or ethnic level, where individuals are generally more adept at recognizing faces from their own racial or ethnic group than from others. The latter is a well-explored phenomenon of the other-race effect (ORE)—adults and children find it difficult to recognize or memorize faces of people from other races [18–25]. However, most research on ORE has focused on individual faces of unfamiliar people from one’s own race and other races. The capacity to recognize multiple images of faces from different races that belong to the same person has been largely overlooked. Laurence & Mondloch [26] hypothesized that people would perceive more identities when completing the sorting task with unfamiliar other-race faces than own-race faces. Their results showed partial evidence that participants made more piles when sorting other-race celebrity faces than their own. Caucasian participants made significantly more misidentification errors when sorting East Asian faces (putting different persons into the same pile); however, this pattern did not appear for East Asian participants sorting Caucasian faces.

Zhou & Mondloch [27] used the card sorting paradigm to explore how individuals perceive and recognize familiar and unfamiliar faces across races. They recruited participants in China to sort greyscale photographs of familiar and unfamiliar celebrities from three racial/ethnic groups (Chinese, Japanese, Caucasian). When sorting unfamiliar faces, participants create more piles for other-race faces than for own-race ones. However, this effect disappeared for familiar faces; participants perfectly grouped familiar faces into two piles, regardless of race or ethnicity. In other words, they demonstrated that identity-specific familiarity (i.e. familiarity at a personal level) surpasses the general challenges in recognizing faces of other races. Latif & Moulson [28] further investigated how familiarity and race affect card sorting with full faces or only internal or external features. They found that participants recognized familiar faces better than unfamiliar ones and created fewer piles for full faces and the internal features than the external features conditions. Regarding race, a further analysis on intrusion errors (i.e. a person reports information that was not among the original materials) revealed that participants made more errors when viewing own-race faces compared with other-race faces, which contradicts the typical findings of the other-race effect. However, Latif & Moulson [28] found no significant difference in recognition performance between own- and other-race faces across all three stimulus conditions. These findings suggest that familiarity at the person level can overcome the general ORE at the race/ethnicity level. When individuals are familiar with faces, regardless of race, they can accurately recognize multiple images of the same person and ‘tell them together’.

Research on within-person face recognition has made significant progress in the last decade. However, most studies primarily focused on Caucasian people sorting own-race faces, with limited studies examining non-Caucasian participants sorting both own- and other-race faces [26–28]. Furthermore, considering the known individual differences in face recognition ability [29,30], previous research has predominantly focused only on a single task of Identity Card Sorting, making it unclear whether individual differences in within-person recognition are associated with broader face-processing skills. To address these knowledge gaps, we examined whether within-person recognition performance correlates with detail-oriented face discrimination abilities and standardized face processing tests, and tested the generalizability of previous findings among Pakistani, Taiwanese and another group of International adults who were neither Taiwanese nor Pakistani.

To this end, we broadened the range of assessment methods by including four tasks to assess different aspects of within-person face recognition. The main task, Face Identity Sorting, had two conditions (Taiwanese and Pakistani) using culturally specific, well-known celebrity photos to manipulate familiarity, measured within-person recognition by asking participants to sort multiple images of the same person despite all the natural variations. We predicted that Taiwanese participants would sort significantly fewer piles for Taiwanese celebrities and more piles for Pakistani celebrities, and vice versa for Pakistani participants. By contrast, we expected international participants would sort more than two piles for both sets of celebrities. The second task was Face Discrimination Task, which involved two conditions (Taiwanese and Pakistani faces). It assessed participants’ deliberate and detail-oriented abilities to detect subtle changes in facial features or configuration, capturing a different aspect of face processing from the memory-based tasks. The third task employed the PI-20 questionnaire [31] to measure subjective difficulties in face recognition. Many studies have reported that people with higher PI-20 scores often struggle to recognize familiar individuals or to match faces seen in different contexts [32–35]. Some studies even found an association between PI-20 and autistic traits in neurotypical adults [36] and adults with autism spectrum disorder (ASD) [37]. Lastly, the Cambridge Face Memory Test (CFMT) [38] was employed to objectively assess each individual’s ability to learn and memorize unfamiliar faces. Together, these tasks allowed us to explore how perceptual discriminability, memory and subjective experience relate to within-person face recognition. By integrating these diverse assessments and extending beyond WEIRD (western, educated, industrialized, rich and democratic) populations, we aim to provide a comprehensive understanding of within-person face recognition and its underlying mechanisms.

## 2. Methods

### 2.1. Participants

A total of 80 adults from three groups joined the study and 73 were retained in the final sample: Taiwanese adults (*n* = 24*,* 11 males, 13 females; *M_age_ =* 22.3, s.d. = 3.2), Pakistan adults (*n* = 25, 15 males, 10 females; *M_age_ =* 27.2, s.d. = 2.6), and International^1^ adults (neither Pakistanis nor Taiwanese) of other countries (*n* = 24, 11 males, 13 females; *M_age_ =* 29.5, s.d. = 7.3). Participants were primarily university students recruited from the Taichung metropolitan area, including China Medical University, Asia University and National Chung-Hsing University. Informed consent was obtained from all participants before the experiment, and the experimental protocols were approved by the Institutional Review Board Committee of China Medical University Hospital, Taiwan. Participants received four tasks at one laboratory visit: A *Face Identity Sorting Task*, a *Face Discrimination Task* and the *PI-20*, and the online *CFMT* in a fixed order. Seven participants’ data were not included in the final sample for various reasons; two Taiwanese participants (both females) were excluded due to their unfamiliarity with both Taiwanese celebrities, and one participant had a PI-20 score higher than 65 (69). Three Pakistani participants (all male) were excluded because they were unfamiliar with both Pakistani celebrities. One International participant (female) was also excluded for being familiar with Taiwanese celebrities, which may have introduced a bias in face recognition performance.

### 2.2. Ethical approval and informed consent statements

The present study adhered to the ethics of scientific publication as detailed in the Principles of Psychologists and the Code of Conduct and to the Committee on Publication Ethics (COPE) guidelines. The Institutional Review Board of the Research Ethics Committee approved the study protocol at China Medical University Hospital, Taichung, Taiwan (Certificate number: CMUH112-REC2-004(CR-1)).

### 2.3. Stimuli and procedures

#### 2.3.1. Face Identity Sorting Task

To manipulate familiarity for different groups of participants, we included two sets of face stimuli (Pakistanis and Taiwanese). Each set contains two well-known celebrities. From Pakistan, we took Azfar Rehman and Danish Taimoor. From Taiwan, we took Bolin Chen and Jay Chou as the stimuli.

**Figure 1.**
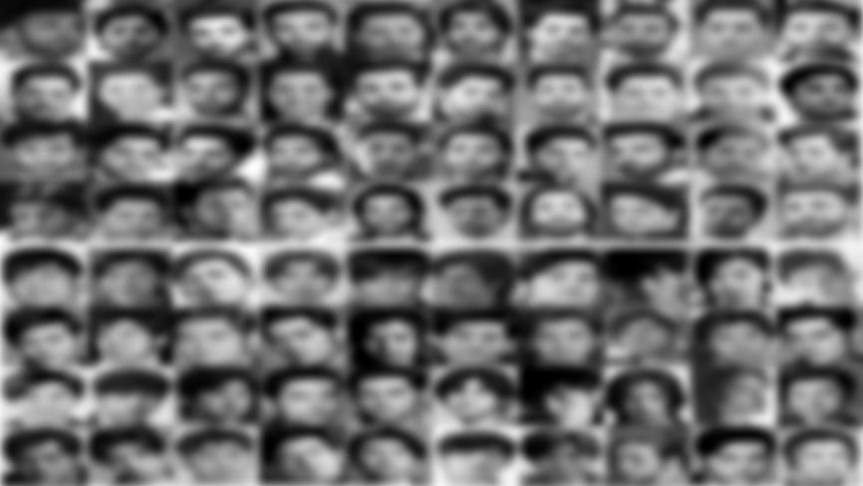
The face stimuli used in the Face Identity Sorting Task. The upper panel shows 40 pictures of two Pakistani celebrities; the lower panel shows 40 pictures of Taiwanese celebrities (note: a blurred version of the stimuli is shown to ensure copyright protection).

*Pakistani celebrities*. This is a familiar condition for the Pakistani participants, whereas for the Taiwanese participants, this is an unfamiliar condition. Twenty pictures of each of the two well-known Pakistani celebrities (Azfar Rehman and Danish Taimoor) served as the stimuli. We downloaded 20 photos from the Internet with a frontal view of each celebrity. Then we selected the photos with a frontal view, face not occluded, some with a bearded face, and some with a shaved face, and had the same size images. These 40 pictures were with various natural expressions (see figure 1). All photos were converted to greyscale images; we used MS PowerPoint to ensure that each image was of equal size, with a width of 1.81 inches and a height of 2.29 inches. The face stimuli were printed out and laminated on A4-sized paper. Each laminated face card was 4.7 cm (width) by 5.7 cm (height) in size. All participants received these 40 photos and were asked to sort them according to their identity (see figure 1).

*Taiwanese celebrities*. This is a familiar condition for Taiwanese participants, whereas for Pakistanis, it is an unfamiliar condition. Likewise, we downloaded 20 pictures of each of the two well-known Taiwanese celebrities, Bolin Chen and Jay Chou, via Google Images from the Internet. These 40 pictures were frontal aspect face photos with varied expressions, hairstyles, viewing angles and illumination. All photos were converted to greyscale images and resized like the Pakistani celebrities. The face stimuli were printed out and laminated on A4-sized paper. Each laminated face card was 4.7 cm (width) by 5.7 cm (height) in size. All participants received these 40 photos and were asked to sort them according to their identity (see figure 1).

*Procedures*. Participants performed the card sorting task in a light grey chamber (90 × 45 × 55 cm) with a uniform background and lighting. Each participant received two conditions, and each condition contained 40 photos to sort. The Taiwanese and Pakistani participants first sorted the unfamiliar condition, followed by the familiar one. For example, Taiwanese participants sorted Pakistani celebrities’ photos first and then Taiwanese celebrities’ photos, while Pakistani participants sorted Taiwanese celebrities’ photos first and then Pakistani celebrities’ photos. For the International participants, both sets of celebrities were unfamiliar. Half of the participants sorted Pakistani celebrities’ photos first, followed by Taiwanese celebrities’; the other half sorted Taiwanese celebrities’ photos, followed by Pakistani celebrities’. Each participant received 40 photos of unfamiliar celebrities, and they were told that ‘you can create multiple groups or you can create a few groups as you think appropriate’. After the participants finished, the experimenter took a picture of the sorted photos facing up and then turned the photos facing down to take a picture from the back. The same procedure was then repeated for the second set. After completing the task, participants were asked whether they knew any of the individuals in the photos. If they said yes, we asked about the name of that person. If they were unfamiliar, they simply said they did not recognize anyone. No additional distractor celebrity faces were included during this screening; the task itself served as the basis for assessing familiarity.

#### 2.3.2. Face Discrimination Task

**Figure 2.**
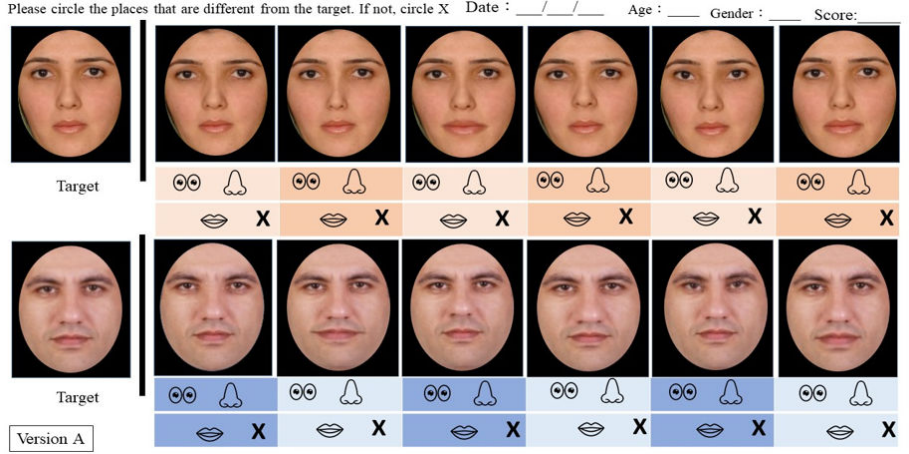
The Face Discrimination Task (The Pakistani version). The leftmost images are the target faces. Participants should circle the specific features (eyes, nose or mouth) in the given options if they notice any changes in those features compared with the target picture. If there is no change, they should circle ‘X’.

The Face Discrimination Task is a paper-and-pencil test, assessing the participant’s ability to detect subtle changes in a face (eyes, nose and mouth). We designed Pakistani and Taiwanese versions. Each version features two target faces, one male and one female, and comprises 12 comparison face images (see figure 2). For the Taiwanese version, the target faces and the 12 altered comparison images were adapted from previous studies from our laboratory [39,40]. For the Pakistani version, two pictures were selected as the target faces. We used the online photo editing application (Photopea, Prague, Czech Republic) to make six alterations for each target face, following the same procedures as the Taiwanese version. Specifically, two alterations were in the eyes (tilting the eyes and widening the space between the two eyes), two in the nose (enlarging the nose by 10%) and elongating the space between the nose and the upper lip, and two in the mouth (enlarging the mouth by 10% and making it smiling). Then, we cropped all these pictures into an oval shape using MS PowerPoint 2016 (Microsoft Co., Redmond, Washington, USA) and printed colour copies of A4-sized paper for distribution to participants. During the task, participants were instructed to carefully examine each image and circle the part of the face where they spotted a change.

#### 2.3.3. Twenty-Item Prosopagnosia Index (PI-20) questionnaire

Each participant received the English version of the PI-20 questionnaire [31] in a slightly modified portrait format [41,42] to evaluate difficulties in face recognition. The questionnaire consists of 20 questions that participants read and respond to based on their experiences, using a five-point scale: 1 for ‘strongly disagree’, 2 for ‘disagree’, 3 for ‘neutral’, 4 for ‘agree’ and 5 for ‘strongly agree’. The score can range from 20 to 100; if someone scores 65 or more, it indicates they may have trouble with face recognition [31]. The PI-20 scores provide a valuable index of participants’ self-reported perspectives on their ability to recognize faces, allowing them to reflect on their personal experiences and perceptions.

#### 2.3.4. The Cambridge Face Memory Test

The CFMT [38] assesses an individual’s ability to remember faces in an online format consisting of four stages: *learning*, *introduction/same images, novel images* and *novel images with noise*. Participants first practise the test procedures before progressing to stages that involve viewing the same images from different angles, encountering new faces with novel orientations and lighting, and identifying faces with added visual noise. In each stage, they quickly memorize six target faces and then identify them among other distractors. The CFMT provides standardized scores measuring face memory performance across different conditions and takes approximately 5 to 10 min to complete.

#### 2.3.5. The measurements for Face Identity Sorting

We calculated three measurements as the key dependent variables, similar to Kramer *et al*. [12]: (i) the number of piles—the total groupings of photos or number of piles created by the participants, (ii) the misidentification errors (MIE)—when participants incorrectly put person A and person B together, we considered it a single misidentification error, and (iii) the sensitivity index d'. The sensitivity index took both the number of piles and the MIE into consideration by measuring participants’ ability to correctly differentiate same-identity and different-identity image pairs.

The sensitivity index d' was calculated based on Balas & Pearson [16] and Kramer *et al*. [12]. For each set of celebrity face stimuli, we first obtained the total number of possible combinations of *same-person pairs* by multiplying the number of distinct identities (2) by the number of ways to choose two items from 20 [202=20×(20-1)(2×1)=20×(19)2=190]. This yielded a value of 380 [ 2×(202)=2×190=380]. Likewise, the total number of possible combinations of the *different identity pairs* was determined by multiplying the combinations of one image of each identity (20 × 20 = 400), which yielded 400 pairs.

False alarms (FA) can be conceptualized as the ‘same person/different group’ errors (i.e. incorrectly categorizing images of the same person separately), which are measured as the proportion of same-identity pairs split into different piles. The calculation involved summing the number of errors from all piles for each participant, then dividing that total by 190 to convert to probability. On the other hand, Hits are defined as Hits = (1 − proportion of misses), where misses are the proportion of different-identity pairs incorrectly assigned to the same pile (i.e. ‘different person/same group’ errors—grouping images of different individuals into the same category). After calculating these two measures (false alarms and hits), we further computed the d' (sensitivity index) using the following formula: d' = z(hits) − z(FA), where z is a z-score. For each stimulus set, we computed the d' for each celebrity and added them together. A higher d' indicates better within-person face recognition (i.e. fewer same-person/different-pile and different-person/same-pile errors).

## 3. Results

### 3.1. Assessments of PI-20 and CFMT

Table 1 summarizes the group characteristics and the group mean scores of PI-20 and CFMT. To test whether Taiwanese, Pakistani and International participants performed equally well on the PI-20 questionnaire, we conducted a one-way ANOVA with *Group* (Taiwanese, Pakistanis and International adults) as the between-subject factor. The main effect of the *Group* was not significant (*p* = 0.371). The mean PI-20 scores were 48.3 (s.e. = 2.4), 44.1 (s.e. = 1.6) and 46.4 (s.e. = 2.3) for Taiwanese, Pakistanis and International adults, respectively. Similarly, for the CFMT, a one-way ANOVA was conducted. The *Group’s* main effect was not significant (*p* = 0.273). The mean CFMT scores were 70.8 (s.e. = 1.3), 75.2 (s.e. = 1.5) and 72.3 (s.e. = 2.8) for Taiwanese, Pakistanis and International adults, respectively.

**Table 1.** Summary of the group characteristics and the mean scores of CFMT and PI-20. PI-20 = Prosopagnosia Index 20 Items, CFMT = Cambridge Face Memory Test.

characteristics	Taiwanese adults (*n* = 24)	Pakistan adults (*n* = 25)	international participants (*n* = 24)	*p*‐value
gender (M : F)	11 : 13	15 : 10	11 : 13	0.528
age (*M* ± s.d.)	22.3 ± 3.2	27.2 ± 2.6	29.5 ± 7.3	<0.001
PI-20 (*M* ± s.d.)	48.3 ± 11.7	44.1 ± 8.0	46.4 ± 11.1	0.371
CFMT (*M* ± s.d.)	70.8 ± 6.3	75.2 ± 7.3	72.2 ± 13.8	0.273
face discrimination (*M* ± s.d.)	16.8 ± 2.8	17.2 ± 3.0	17.3 ± 2.6	0.275

### 3.2. Performance on Face Discrimination Task

**Figure 3.**
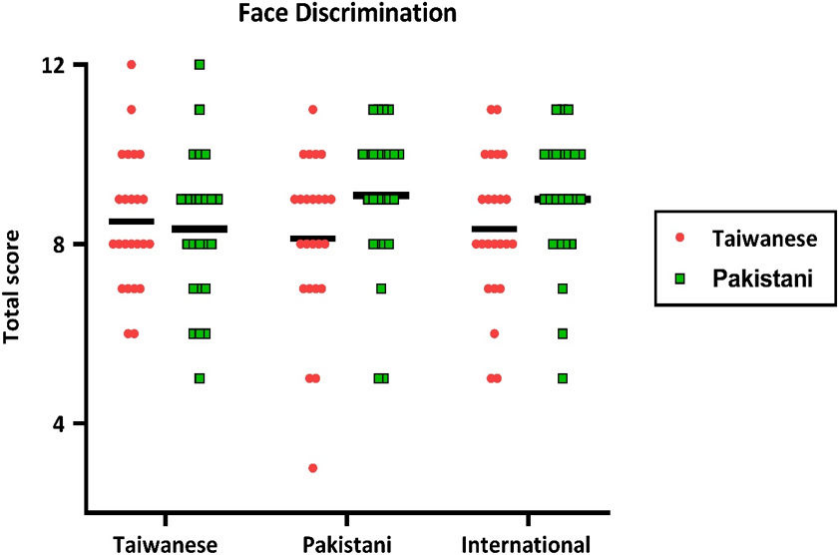
The main results of the Face Discrimination Task. The individual’s and group mean scores are shown for the two stimulus conditions (red represents the Taiwanese version and green represents the Pakistani version). The Y-axis represents the score of face discrimination (full score is 12), and the X-axis shows the three groups. Each dot corresponds to one individual’s data; the black horizontal bar indicates the group mean.

We ran a two-way mixed ANOVA with *Group* (Taiwanese, Pakistanis and International adults) as the between-subject factor and *Face Type* (Taiwanese and Pakistanis) as the within-subject factor. The main effect of the *Group* was not significant (*p* = 0.275). For Taiwanese participants, the mean score was 8.42 ± 1.58, for Pakistani participants, it was 8.62 ± 1.77, and for International participants, it was 8.67 ± 1.59. The main effect of *Face Type* was significant, *F*(1,70) = 9.1, *p* = 0.003, *η_p_*^2^ = 0.116. The mean scores for the Pakistani face version (8.86, s.d. = 1.54) were significantly greater than the Taiwanese face version (8.27, s.d. = 1.69). The interaction effect was not significant (*p* = 0.808). Figure 3 illustrates the score of the face discrimination of the two conditions for the three groups.

### 3.3. Performance on Face Identity Sorting

To ensure valid manipulation of culture-specific familiarity, we included only those participants who knew at least one celebrity from their own country. Among the 24 Taiwanese participants, all knew Jay Chou, while 18 recognized Bolin Chen; none knew Pakistani celebrities Danish Taimoor or Azfer Rehman. Of the 25 Pakistani participants, 23 knew Danish Taimoor and Azfer Rehman, while none recognized Bolin Chen and Jay Chou. All 24 International participants were unfamiliar with both Taiwanese and Pakistani celebrities. Three measurements were used as the key dependent variables: (i) the number of piles, (ii) MIE, and (iii) the sensitivity index (d').

### 3.4. Number of piles

**Figure 4.**
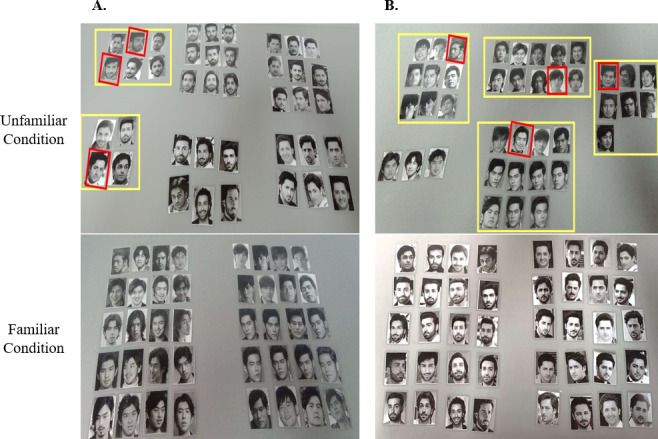
Illustration of sample results of the face sorting task, featuring a representative Taiwanese (Panel A) and a representative Pakistani participant (Panel B). In each panel, the top row displays the unfamiliar condition, while the bottom row displays the familiar condition. The yellow squares highlight examples of different identity pairs (photos of distinct individuals sorted into the same pile), with the red squares indicating examples of a misidentified person within that pile. The remaining non-framed piles represent examples of the same identity pairs (photos of the same individual sorted into the same pile).

**Figure 5.**
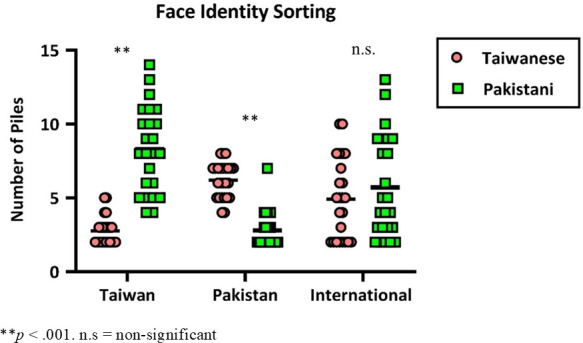
The number of sorted piles of the two stimulus conditions (Taiwanese celebrity in red, Pakistani celebrity in green) for the three groups. The Y-axis represents the number of piles, while the X-axis shows the three groups. Each dot corresponds to one individual’s data; the black horizontal bars indicate the group mean.

Figure 4 illustrates two representative results of identity sorting (including the unfamiliar and the familiar conditions) from a Taiwanese and a Pakistani participant. For the Taiwanese group, the mean number of identities perceived for Taiwanese stimuli (the familiar condition) was 2.8, s.d. = 0.9, *R* = 3. A one-sample *t*‐test confirmed this was significantly higher than the two identities presented (*t*(23) = 3.89, *p* = 0.0009). For Pakistani stimuli (the unfamiliar condition), the mean number of sorted identities was 8.3, s.d. = 2.9. *R* = 10, significantly higher than the two identities presented (*t*(23) = 3.2, *p* = 0.0044). Moreover, a two-sample *t*‐test comparing the familiar (Taiwanese celebrities) and unfamiliar conditions (Pakistani celebrities) revealed a significant difference between the two conditions (*t*(46) = 8.91, *p* < 0.001). Figure 5 illustrates the number of sorted piles of the two stimulus conditions for the three groups.

For the Pakistani group, the mean number of identities perceived for Taiwanese stimuli (the unfamiliar condition) was 6.4, s.d. = 1.8, *R* = 8, significantly higher than the two identities presented (*t*(24) = 11.76, *p* < 0.001). For Pakistani stimuli (the familiar condition), the mean was 2.8, s.d. *=* 1.2, *R* = 5, also significantly higher than the two identities presented (*t*(23) = 10.64, *p* < 0.001). A two-sample *t*‐test revealed a significant difference between the two conditions (*t*(48) = 8.32, *p* < 0.001). For the International group, the mean number of identities perceived for Taiwanese stimuli (unfamiliar condition) was 4.9, s.d. = 2.7, *R* = 8, significantly higher than the two identities presented (*t*(23) = 5.25, *p* < 0.001). For Pakistani stimuli, the mean was 5.7, s.d. = 3.4, *R* = 11, also significantly higher than the two identities presented (*t*(23) = 5.27, *p* < 0.001). A two-sample *t*‐test comparing both conditions revealed no significant difference (*p* = 0.381).

### 3.5. Misidentification errors

**Figure 6.**
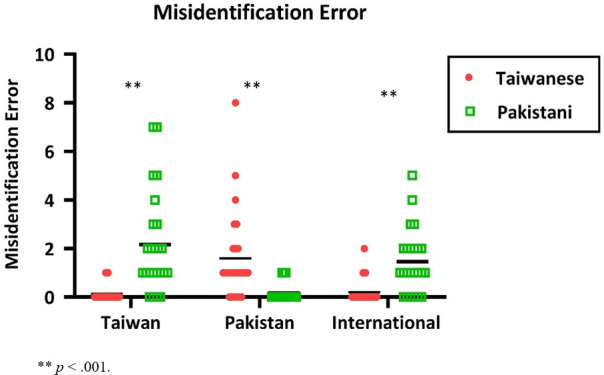
The number of misidentification errors of the two stimulus conditions (red colour is for the Taiwanese stimuli; green colour is for the Pakistani stimuli) for the three groups. The Y-axis represents the number of misidentification errors, while the X-axis shows the three groups. Each dot corresponds to one individual’s data; the black horizontal bars indicate the group mean.

Figure 6 illustrates the group mean numbers of misidentification errors of the two stimulus conditions. For the Taiwanese participants, the mean number of Misidentification errors for Taiwanese stimuli (the familiar condition) was 0.1, s.d. = 0.3, which was not significantly different from zero (*t*(23) = 1.8, *p* = 0.07). For Pakistani stimuli (the unfamiliar condition), the mean number of Misidentification errors was 2.2, s.d. = 2.0, significantly higher than zero (*t*(23) = 4.2, *p* < 0.001). Moreover, a two-sample *t*-test revealed that Pakistani stimuli yielded significantly more Misidentification errors than Taiwanese stimuli (*t*(46) = 8.91, *p* < 0.001).

For the Pakistani participants, the mean number of Misidentification errors for Pakistani stimuli (the familiar condition) was 0.2, s.d. = 0.4. A one-sample *t*‐test confirmed it is significantly higher than zero (*t*(24) = 2.1, *p* = 0.05). For Taiwanese stimuli (the unfamiliar condition), the mean number of Misidentification errors was 1.6, s.d. = 1.8, significantly higher than zero, (*t*(24) = 4.2, *p* < 0.001). A two-sample *t*‐test showed that the Taiwanese stimuli yielded significantly more Misidentification errors than Pakistani stimuli (*t*(48) = −3.6, *p* < 0.001).

The International participants’ mean number of Misidentification errors for Pakistani stimuli was 1.5, s.d. = 1.3. A one-sample *t*‐test confirmed it is significantly higher than zero (*t*(23) = 5.7, *p* = 0.001). For Taiwanese stimuli, the mean number of Misidentification errors was 0.2, s.d. = 0.5. A one-sample *t*‐test (*t*(23) = 2.0, *p* = 0.057) showed that it is marginally significant. Moreover, a two-sample *t*‐test comparing the two conditions showed that the Pakistani stimuli yielded significantly more Misidentification errors than the Taiwanese stimuli (*t*(46) = −4.36, *p* < 0.001).

### 3.6. Sensitivity measure

To test whether the Taiwanese, Pakistani and International participants performed equally well on the Face Identity Sorting, we ran a two-way mixed ANOVA with *Group* (Taiwanese, Pakistanis and International adults) as the between-subject factor and *Stimuli Type* (Taiwanese and Pakistanis) as the within-subject factor on the sensitivity index (d'). The *Group* main effect was not significant (*p* = 0.785). The mean d's for the Taiwanese, Pakistanis and International participants were 2.359 (s.e. = 0.165), 2.480 (s.e. = 0.161) and 2.328 (s.e. = 0.165), respectively. The main effect of the *Stimulus Condition* was significant, *F*(1,70) = 6.71, *p* = 0.012 *η_p_*^2^ = 0.088. The mean d' for the Taiwanese celebrity (*M* = 2.58, s.e. = 0.13) was greater than that of the Pakistani celebrity (*M* = 2.19, s.e. = 0.10).

**Figure 7.**
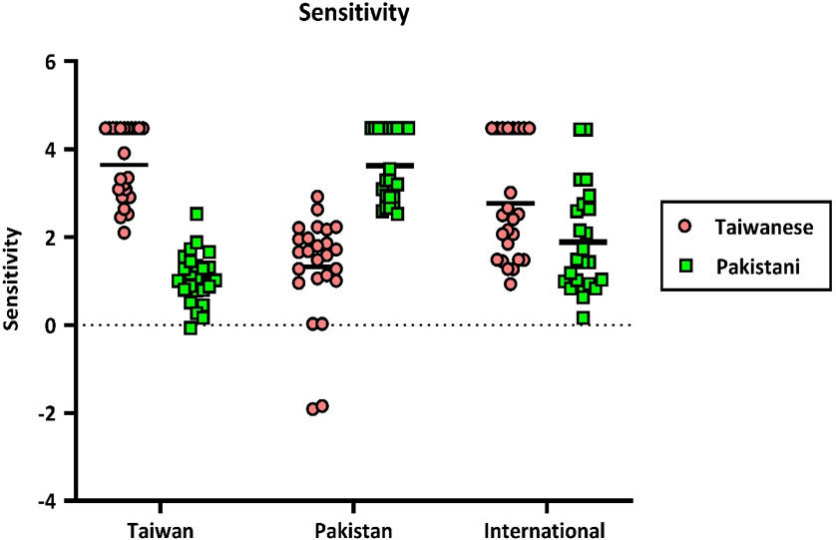
The sensitivity score (d') of the two stimulus conditions (pink colour for the Taiwanese stimuli and green colour for the Pakistani stimuli) for the three groups. The Y-axis represents the sensitivity score, while the X-axis shows the three groups. Each dot corresponds to one individual, the black horizontal bars indicate the group mean.

Importantly, there was a significant interaction effect between *Stimulus Condition* and *Group*, *F*(2,67) = 90.63, *p* < 0.001, *η_p_*^2^ = 0.730, indicating that the effect of *Stimulus Condition* varied across the three groups. Further analyses on the simple main effect of *Stimulus Condition* for each group revealed that in the Taiwanese group, the d' for Taiwanese celebrities (*M* = 3.64, s.e. = 0.23) was higher than for Pakistani celebrities (*M* = 1.07, s.e. = 0.18) (*p* < 0.001). For the Pakistani group, the d' for Pakistani celebrities (*M* = 3.63, s.e. = 0.17) was higher than for Taiwanese celebrities (*M* = 1.32, s.e. = 0.22) (*p* < 0.001). For the International group, the d' for Taiwanese celebrities (*M* = 2.77, s.e. = 0.23) is higher than for Pakistani celebrities (*M* = 1.88, s.e. = 0.18) (*p* = 0.019). Figure 7 illustrates the sensitivity of the two stimulus conditions for the three groups.

### 3.7. Correlation and regression analyses

To examine whether an individual’s within-person face recognition performance correlated with face discrimination ability and standardized face recognition tasks, we conducted a series of bivariate correlation analyses. Specifically, we examined the associations among Face Identity Sorting (using the d' sensitivity index), PI-20, CFMT and Face Discrimination separately for the three groups: Taiwanese (table 2), Pakistanis (table 3) and International participants (table 4). We also analysed these associations across all participants combined. The correlations and *p*-values for all participants combined are summarized in table 5.

**Table 2.** The correlations among the PI-20, CFMT, d' (Tw), d' (Pk), d' (sum), Face Discrimination (Tw), Face Discrimination (Pk) and Face Discrimination (sum) in Taiwanese participants. CFMT = Cambridge Face Memory Test, d' = the sensitivity measures in the Face Identity Sorting, FD = Face Discrimination, Pk = Pakistan, Tw = Taiwan.

	PI-20	CFMT	d' (Tw)	d' (Pk)	d' (sum)	FD (Tw)	FD (Pk)	FD (sum)
PI-20	—	0.130 (0.544)	−0.167 (0.436)	−0.244 (0.250)	−0.238 (0.262)	0.367 (0.077)	0.253 (0.232)	0.356 (0.088)
CFMT	—	—	0.296 (0.160)	0.214 (0.315)	0.315 (0.134)	0.450 (0.025)	0.324 (0.122)	0.447 (0.029)
d' (Tw)	—	—	—	0.362 (0.082)	891^**^ (<0.001)	0.195 (0.361)	0.043 (0.841)	0.140 (0.513)
d' (Pk)	—	—	—	—	0.746^**^ (<0.001)	0.407 (0.048)	0.155 (0.469)	0.328 (0.118)
d' (sum)	—	—	—	—	—	0.338 (0.106)	0.107 (0.620)	0.260 (0.219)
FD (Tw)	—	—	—	—	—	—	0.549^*^ (0.005)	0.894^**^ (<0.001)
FD (Pk)	—	—	—	—	—	—	—	0.865^**^ (<.001)
FD (sum)	—	—	—	—	—	—	—	—

*p < 0.01; **, *p* < 0.001.

**Table 3.** The correlations among the PI-20, CFMT, d' (Tw), d' (Pk), d' (sum), Face Discrimination (Tw), Face Discrimination (Pk) and Face Discrimination (sum) in Pakistani participants. CFMT = Cambridge Face Memory Test, d' = the sensitivity measures in the Face Identity Sorting, FD = Face Discrimination, Pk = Pakistan, Tw = Taiwan.

	PI-20	CFMT	d' (Tw)	d' (Pk)	d' (sum)	FD (Tw)	FD (Pk)	FD (sum)
PI-20	—	0.113 (0.592)	0.072 (0.733)	−0.272 (0.188)	−0.094 (0.656)	−0.349 (0.088)	−0.531^*^ (0.006)	−0.495 (0.012)
CFMT	—	—	0.442 (0.027)	0.202 (0.333)	0.476 (0.016)	−0.169 (0.420)	−0.347 (0.089)	−0.288 (0.163)
d' (Tw)	—	—	—	0.012 (0.955)	0.829^**^ (<0.001)	−0.174 (0.404)	−0.068 (0.746)	−0.141 (0.500)
d' (Pk)	—	—	—	—	0.569^*^ (0.003)	0.334 (0.102)	0.252 (0.224)	0.336 (0.100)
d' (sum)	—	—	—	—	—	0.044 (0.835)	0.085 (0.686)	0.072 (0.732)
FD (Tw)	—	—	—	—	—	—	0.543^*^ (0.005)	0.892^**^ (<0.001)
FD (Pk)	—	—	—	—	—	—	—	0.864^**^ (<.001)
FD (sum)	—	—	—	—	—	—	—	—

*p < 0.01; **p < 0.001.

**Table 4.** The correlations among the PI-20, CFMT, d' (Tw), d' (Pk), d' (sum), Face Discrimination (Tw), Face Discrimination (Pk) and Face Discrimination (sum) in International participants. CFMT = Cambridge Face Memory Test, d' = the sensitivity measures in the Face Identity Sorting, FD = Face Discrimination, Pk = Pakistan, Tw = Taiwan.

	PI-20	CFMT	d' (Tw)	d' (Pk)	d' (sum)	FD (Tw)	FD (Pk)	FD (sum)
PI-20	—	−0.146 (0.497)	−0.310 (0.140)	−0.109 (0.612)	−0.261 (0.219)	−0.383 (0.065)	−0.117 (587)	−.308 (0.143)
CFMT	—	—	0.520^*^ (0.009)	0.712^**^ (<0.001)	0.737^**^ (<0.001)	0.099 (0.645)	0.499 (0.013)	0.351 (0.092)
d' (Tw)	—	—	—	0.369 (0.076)	0.848^**^ (<0.001)	0.071 (0.743)	0.088 (684)	0.095 (0.658)
d' (Pk)	—	—	—	—	0.806^**^ (<0.001)	0.312 (137)	0.486 (0.016)	0.477 (0.018)
d' (sum)	—	—	—	—	—	0.224 (0.293)	0.333 (0.112)	0.333 (0.112)
FD (Tw)	—	—	—	—	—	—	0.372 (0.074)	0.844^**^ (<0.001)
FD (Pk)	—	—	—	—	—	—	—	0.812^**^ (<.001)
FD (sum)	—	—	—	—	—	—	—	—

*p < 0.01; ** *p* < 0.001.

**Table 5.** The correlations among the PI-20, CFMT, d' and Face Discrimination task. CFMT = Cambridge Face Memory Test, d' = the sensitivity measures in the Face Identity Sorting, FD = Face Discrimination.

	PI-20	CFMT	d' (sum)	FD (sum)
PI-20	—	−0.050 (0.337)	−0.214 (0.035)	−0.117 (0.162)
CFMT	—	—	0.605^**^ (<0.001)	0.173 (0.071)
d' (sum)	—	—	—	0.217 (0.033)
FD (sum)	—	—	—	—

*p < 0.01; **p < 0.001.

Table 2 summarizes the bivariate correlations among the four tasks for the Taiwanese participants. The CFMT showed a trend of positive correlation with Face Discrimination for the Taiwanese version (Tw) (*r* = 0.456, *p* = 0.025) and for the summed score (*r* = 0.447, *p* = 0.029). However, neither correlation reached statistical significance with an adjusted alpha value of 0.0017 (= 0.05/28, accounting for multiple comparisons). Pakistanis’ celebrity Face Identity Sorting (d' (Pk)), which was an unfamiliar condition for Taiwanese participants, showed a trend of positive correlation with the Taiwanese version of the Face Discrimination Task (*r* = 0.407, *p* = 0.048). However, it was not significant with an adjusted alpha value of 0.0017.

Table 3 summarizes the bivariate correlations among the four tasks for the Pakistani participants. The PI-20 showed a trend of negative correlation with the Pakistanis version of the Face Discrimination task (*r* = −0.531, *p* = 0.006) and the summed scores of Face Discrimination (*r* = −0.495, *p* = 0.012). However, neither correlation value reached statistical significance with an adjusted alpha value of 0.0017 (= 0.05/28). For the CFMT, we found a trend of positive correlation with the Taiwanese celebrity Face Identity Sorting (d' (Tw)) (*r* = 0.442, *p* = 0.027), which is the unfamiliar condition, and with the summed Face Identity Sorting (d' (sum)) (*r* = 0.476, *p* = 0.016). However, neither correlation value reached statistical significance with an adjusted alpha value of 0.0017 (= 0.05/28).

Table 4 summarizes the bivariate correlations among the four tasks for the International participants. With an adjusted alpha value of 0.0017 (= 0.05/28), the CFMT marginally correlated with the Taiwanese celebrities’ Face Identity Sorting (d' (Tw)) (*r* = 0.520, *p* = 0.009), positively correlated with the Pakistani celebrities’ Face Identity Sorting (d' (Pk)) (*r* = 0.712, *p* < 0.001) and the summed Face Identity Sorting (d' (sum)) (*r* = 0.737, *p* < 0.001). Moreover, CFMT showed a trend of positive correlation with the Pakistani version of the Face Discrimination Task (*r* = 0.499, *p* = 0.013), but it was not statistically significant.

Lastly, to gain an overall insight into the pattern of correlations across all participants, we also conducted bivariate correlations among tasks using combined total scores. Table 5 summarizes the correlations among the four tasks across 73 participants. With an adjusted alpha value of 0.0083 (= 0.05/6), we observed one important correlation, that is, an individual’s d' significantly correlated with CFMT scores (*r* = 0.605, *p* < 0.001). Although an individual’s d' showed a trend of negative correlation with PI-20 score (*r*= −0.214, *p* = 0.035) and a trend of positive correlation with Face Discrimination (*r* = 0.217, *p =* 0.033), the correlational strength did not reach statistical significance.

In addition to correlational analyses, we further conducted a multiple regression analysis to assess whether PI-20, CFMT and Face Discrimination scores could predict performance on the Face Identity Sorting task (d') for all participants. We used PI-20, CFMT and Face Discrimination (Sum) to serve as predictors (independent variables), while Face Identity Sorting (d') performance was the outcome variable. Table 6 summarizes the results of multiple regression analysis. We first performed a collinearity diagnosis for the three predictors. The tolerance values for PI-20, CFMT and Face Discrimination were 0.985, 0.969 and 0.958, respectively. This suggests that the three predictors were not correlated with one another and contributed unique variance to the outcome. The overall model was significant, *R²* = 0.408, *F*(3, 69) = 15.88, *p* < .001. Among the predictors, CFMT was a significant predictor, *b* = 0.094, (95% CI .064, .125), *t*(69) = 6.16, *p* < 0.001, suggesting that better face memory is associated with improved within-person face recognition performance. PI-20 was a marginally significant predictor, *b* = –0.027, (95% CI –0.055, 0.022), *t*(69) = –1.86, *p* = 0.067, indicating that self-rated face recognition difficulties are linked to lower d' scores.

**Table 6.** Multiple regression analysis predicting Face Identity Card Sorting performance (d') from PI-20, CFMT and Face Discrimination scores (*n* = 73). B = unstandardized beta, *b* = standardized beta, PI-20 = Prosopagnosic Index 20-items, CFMT = Cambridge Face Memory Test, Face Disc (sum) = Face Discrimination (sum).

variables	B	*b*	*p*
Constant	−1.811		0.246
PI-20	−0.027	−0.173	0.067
CFMT	0.094	0.579	0.001
Face Disc (sum)	0.055	0.096	0.313
R^2^		0.408	
F		15.877**	

**p <.001,* p <.01

## 4. General discussions

This study investigated within-person face recognition in Taiwanese, Pakistanis and international adults using a Face Identity Sorting task (using photos of Taiwanese and Pakistani celebrities as the stimuli), a Face Discrimination task (two conditions), a self-report questionnaire (PI-20) and an online computerized face memory test (CFMT). We explored the associations among an individual’s within-person face recognition, face discrimination, self-reported face processing ability and face memory performance. The results revealed several noteworthy findings. First and foremost, we observed a robust effect of familiarity for both Taiwanese and Pakistani adults, in which recognizing celebrities from their own country was more accurate. By contrast, international adults unfamiliar with both Taiwanese and Pakistani stimuli exhibited significantly lower sensitivity to both sets of stimuli, replicating the robust effect of familiarity^2^ found in previous studies involving Caucasian participants. For the Face Discrimination task, CFMT and PI-20, all three groups performed equally well. This indicates that the three samples of participants have roughly equal abilities in objectively measured face processing skills such as featural/configural processing and face memory, as well as subjectively self-rated proficiency in face recognition. Most importantly, the correlation analysis suggests that the sensitivity index (d') for the identity sorting task (which reflects the participant’s ability to correctly identify the same person’s face images aside from distractors) positively correlates with CFMT and exhibited a trend of negative correlation with PI-20, meaning that individuals with better within-person recognition also have better face memory and tended to report less face recognition problems. In addition, an individual’s d' also exhibited a trend of positive correlation with Face Discrimination, indicating that those who excel in within-person recognition were more likely to have better detail-oriented abilities in detecting small alterations in facial features (i.e. widening eyes, large nose and a widening mouth). The following discussions will elaborate on the present study’s results and limitations.

### 4.1. Our results extend the previous findings in the Asian context

In the results of the Face Identity Sorting Task, we found a significant familiarity effect, meaning that individuals who were familiar with the celebrities sorted them into significantly fewer piles (i.e. close to two) than those who were unfamiliar with the celebrities. Conversely, international participants who were unfamiliar with both sets of celebrities sorted into more than two piles in both conditions. Overall, the results of our within-subject design study are similar to Jenkin *et al*.’s [5] study, which used a between-subjects design. They used photos of two Dutch celebrities and tested UK and Dutch participants. The UK undergraduate students unfamiliar with the two Dutch celebrities tended to falsely divide a single person into several identities; however, Dutch undergraduate students who were familiar with the two celebrities sorted them into two piles perfectly. They also demonstrated that familiarity with a face significantly influences how well we can accommodate within-person variability by demonstrating very rare misidentification errors, which agrees with our observation that there were significantly fewer misidentification errors when participants were familiar with the celebrities.

Notably, unlike Jenkins *et al.* [5], we used a within-subjects design and started with the unfamiliar conditions, followed by the familiar ones. This fixed test order is designed to strategically minimize the chance that participants may spontaneously realize there were only two identities. This within-subject design seemed effective and did not induce the practice effect due to test order. This is supported by the sorting results of the participants of the International Group, who confirmed that familiarity is still important, as they did not do the same as the Taiwanese and Pakistani participants. Moreover, the test order for the International participants was randomized to prevent potential order effects. Half of the participants started with the Taiwanese set of stimuli, followed by the Pakistani set, while the other half started with the Pakistani set, followed by the Taiwanese set. We did not find any evidence indicating that the number of sorted piles for the second set of stimuli was significantly lower than for the first set of stimuli. Our results also generally agree with the findings of Kramer *et al*. [12], who used two sets of celebrity face stimuli and computed the number of sorted piles as well as the sensitivity index d', which was closer to our experimental design. In their study, one set comprised two Dutch female celebrities, and another set was made up of two British male celebrities, with 40 photos per set. They also manipulated three presentation conditions: full-face, internal and external features. They found high sensitivity and a familiarity effect for familiar faces in the full-face and internal feature conditions by computing the number of piles and sensitivity measures. Our results align well with theirs, as we also found high sensitivity (d') for familiar faces in Taiwanese and Pakistani participants.

As we expected, international participants were unfamiliar with Taiwanese and Pakistani celebrities, which led them to sort significantly more than two piles in each condition. However, we did observe that face stimuli of Taiwanese celebrities appeared to be easier than those of Pakistani celebrities, as the mean number of sorted identities of the former (*M* = 4.9, s.d. = 2.7) was significantly less than that of the latter (*M* = 5.7, s.d. = 3.4) (*p* < 0.001). This may be due to two possibilities. First, it could be that the physical similarity between Taiwanese celebrities and Pakistani celebrities was unequal. Among the Taiwanese celebrities, Jay Chou has small eyes and Bolin Chen has big eyes, while the Pakistani celebrities both have big eyes. Second, although the international participants are not familiar with Taiwanese celebrities, having stayed in Taiwan for more than six months, they are generally more familiar with the overall facial features of the Taiwanese people.

### 4.2. Within-person face recognition and detail-oriented face discrimination

In this study, we employed a custom-made Face Discrimination Task to assess participants’ ability to detect subtle changes in facial features (i.e. making the nose bigger or raising the corners of the mouth) or configural information between features (i.e. widening the space between the eyes). We observed comparable performance across all three groups, with no significant own-race advantage or other-race effects. It is worth noting that across all participants, our results (shown in table 5) revealed a trend of positive correlation between d' (sensitivity index) and the total scores of the Face Discrimination Task, indicating that individuals who are better at detecting small changes in the eyes, nose and mouth on a face were more likely to perform better in within-person face recognition. In other words, there may be an association between within-person face recognition performance and proficiency in detail-oriented face discrimination (i.e. the ability to detect subtle changes in facial features or configuration) when considering all participants across the groups.

The observation that the ability to detect subtle changes in facial features or configuration may correlate with within-person face recognition performance generally agrees with a recent study by Logan *et al*. [43]. They used digitally manipulated faces to investigate how well people could distinguish between faces in three conditions: full faces, individual features (e.g. nose, eyes) and feature groups (internal: eyes, nose, mouth; or external: head shape, hairline). They found that sensitivity to internal facial features (eyes, nose and mouth) was comparable to the sensitivity of the most diagnostic individual features, highlighting that while internal features are essential for face processing, the overall contribution of internal features is limited by the most informative single feature. Our findings also align with a recent study by Leong *et al*. [44], who investigated the role of featural and configural processing in face memory, assessed by the Old/New Recognition Memory Task and the Chinese version of the Cambridge Face Memory Test (CFMT-Chi). They found that both holistic and featural processing play important roles in face recognition memory, with featural processing being particularly significant. Our study also shows a trend that individuals who excel in detecting subtle changes in the eyes, nose or mouth in a face tend to perform better in within-person face recognition.

### 4.3. Within-person face recognition, Cambridge Face Memory Test and PI-20 performance

In this study, we initially conducted separate correlational analyses for Taiwanese, Pakistani and International participants to understand the relationships between within-person face recognition, the CFMT and the PI-20. This approach allowed us to assess the patterns of association within each individual group. Following this, we conducted a combined correlation analysis across all three groups to explore the broader trends and identify links among within-person face recognition, CFMT and PI-20.

We found that the CFMT score appears to correlate more closely with Identity Sorting performance when it is presented in an unfamiliar condition. For example, for the International participants who were unfamiliar with both Taiwanese and Pakistani celebrities, their CFMT scores significantly correlated with the Taiwanese celebrities’ face identity sorting (d' (Tw)) and Pakistani celebrities’ face identity sorting (d' (Pk)), demonstrating that individuals with better face memory tend to show better within-person recognition of unfamiliar faces. However, it is important to note that the observed pattern of correlation was not symmetric among the three groups of participants. In the case of Taiwanese participants, the CFMT showed a non-significant positive correlation (*r* = 0.214, *p* = 0.315) with Face Identity Sorting for Pakistani faces (i.e. the unfamiliar condition). Similarly, among Pakistani participants, we observed a trend of positive correlation with the Taiwanese celebrity Face Identity Sorting (d' (Tw)) (*r* = 0.442, *p* = 0.027), which is the unfamiliar condition, but the correlation did not reach significance with the adjusted alpha level of 0.0017 (= 0.05/28). We found ourselves somewhat perplexed by the absence of a significant correlation. This asymmetry suggests that the relationship between face memory and within-person face recognition may be affected by additional factors that need further exploration.

More importantly, this association was again observed between the d' (sum) in the Face Identity Sorting task, which measures overall sensitivity in within-person face recognition, and positively correlates with CFMT when all three groups of participants were combined. Why does CFMT correlate with within-person recognition? The CFMT is designed as a memory-based test that evaluates how well people can recognize unfamiliar faces by building representations after a brief learning period. The test structure of the CFMT enables it to effectively measure both between-person (i.e. ‘telling people apart’) and within-person recognition (i.e. ‘telling people together’—identifying the same individual across different images), which may contribute to the observed correlation. Regarding the aspect of between-person comparison, participants must memorize six different target faces during the learning stage, enabling the assessment of variability in face memory ability among individuals. Regarding the within-person comparisons, it manifests as participants progress through increasingly challenging tasks, such as viewing the same images, novel images and novel images with noise, which indicates how an individual’s performance adapts to changes in pose, illumination and image quality of each learnt target face. These stages test the strength of face recognition mechanisms by challenging participants to move beyond image-based recognition and rely on robust three-dimensional face-representation processes. The novel images and novel images with noise stages are crucial as they assess how effectively participants can recognize faces under challenging conditions, such as varying poses or added noise. This underscores individual adaptability and differences in face recognition. It also reflects a similar underlying mechanism involved in within-person face recognition.

Additionally, we observed a negative correlation trend across all participants between the d' (sum) in the Face Identity Sorting task and the Prosopagnosia Index (PI-20) score, a 20-item self-report questionnaire designed to assess difficulties in face recognition. This may suggest that individuals who reported fewer face recognition difficulties (i.e. having a lower PI-20 score) tended to perform better in the Face Identity Sorting task. In practical terms, participants with lower self-reported trouble with faces were better able to distinguish ambient photos of the same person across varying appearances, indicating stronger within-person face recognition abilities. Within-person recognition relies on the ability to generalize across natural variations in a person’s appearance (e.g. different photos, expressions and viewing angles). Indeed, PI-20 is designed to capture subjective experiences of face recognition difficulty in real-life situations. Several studies have reported that individuals with higher PI-20 scores tend to have difficulties in recognizing familiar faces or matching faces seen in different contexts [32–35]. Our findings have revealed a potential link between within-person face recognition and the self-rated PI-20 score.

### 4.4. Conclusion, limitations and future work

This study explored within-person face recognition in the context of Pakistani and Taiwanese adults, examining within-person face recognition using Face Identity Sorting, Face Discrimination, CFMT and PI-20. We found a significant familiarity effect in Face Identity Sorting. With all participants combined, we found that Face Identity Sorting performance (in terms of d' sensitivity) can be predicted by the CFMT scores, and showed mild associations with Face Discrimination and PI-20 scores, suggesting that overall, individuals who have better face memory, are good at detecting subtle changes in the eyes, nose and mouth, and reported less difficulties with faces, performed generally better in within-person face recognition. These findings highlight that better face memory and detail-oriented discrimination abilities are associated with enhanced within-person face recognition.

This study has several limitations. One limitation is the age difference among the three groups. On average, the Pakistanis and International participants were older than the Taiwanese participants. Moreover, because our participants are foreign students, there is an unequal amount of exposure to Taiwanese and Pakistani faces among the three groups. Namely, all Pakistanis and International participants in this study live in Taiwan and have been exposed to many Taiwanese faces. By contrast, the Taiwanese participants had limited exposure to Pakistani faces. These unequal ages and exposure to the unfamiliar type of face may account for some of the imperfect, asymmetrical findings we observed in the study. Another limitation is that the PI-20 was administered right after the Face Identity Sorting, which may have influenced participants’ self-evaluations by reflecting on their previous performance, possibly inflating the correlations between the PI-20 and the face tests.

Future research shall consider changing the order of the tasks and administering the subjective questionnaire before the main tasks. Additionally, we would like to conduct a two-by-two design to test both Taiwanese and Pakistani faces under familiar and unfamiliar conditions to further investigate the identity-specific and race/ethnicity level of familiarity effect. The present study extended beyond WEIRD populations and integrated diverse assessments to address how perceptual discriminability, memory and subjective experience relate to within-person face recognition. Our findings contributed to a more comprehensive understanding of within-person face recognition and its underlying mechanisms.

## Data Availability

The original datasets (as an Excel file) for the three groups have been made publicly available and stored at Open Science Framework [45].

## Footnotes

^1^
The International participants who were not Pakistani, consist of 14 students from Asia (1 female from the Philippines, 3 males and 2 females from Vietnam, 2 males and 3 females from Indonesia, 1 female from Thailand, 1 male from Sri Lanka, 1 male from Myanmar), 2 students from Europe (1 female from Austria, 1 male from Germany), 3 students from Africa (1 female from Swaziland (Eswatini), 1 male from Malawi, 1 male from Rwanda), and 5 students from North and South America (1 male and 1 female from USA, 1 female from Mexico, 1 female from Bolivia, and 1 female from Venezuela).

^2^
We were aware that our familiarity effect may result from a combination of identity-specific familiarity (personal level of familiarity) and ethnicity-based familiarity (i.e. the other-race effect). Both factors operate in a similar direction within one’s own race and with personally familiar celebrities. The identity-specific familiarity at a personal level trumps the general small disadvantage of discriminating among faces of other-race individuals, as suggested by Zhou & Mondloch [27] and Latif & Moulson [28]. For example, we can recognize different face images of Bill Clinton without a problem. This is because we have a personal-level familiarity with him even though his face would be considered as other-race example for most Asian people. In other words, here we treat the effect of familiarity at the identity-specific level and rather than at the ethnicity-based level.
